# Latent profiles of the perceived age-friendliness of cities and communities in Colombia: relations to ageism experiences in later life

**DOI:** 10.1186/s12877-026-07735-w

**Published:** 2026-07-29

**Authors:** Xuefei Li, Marcela Bustamante, Nelson Andrés Tamayo Gaviria, Deinedt Castellanos, Álvaro Pío Jaramillo, Gina Paola Brochero, Elizabeth Fajardo Ramos, Estella Melguizo Herrera, Norma Yasmith Martínez Vargas, Juandavid Romero Escudero, Maya Abi Chahine, Aidan Timlin, Annie Wright, Aja Louise Murray

**Affiliations:** 1 Department of Psychology, University of Edinburgh, Edinburgh, UK; 2 HelpAge International, London, England; 3 Colombian Network for Dignified and Active Ageing (RedCoenve), Bogotá, Colombia; 4 Faculty of Law and Political Science, Los Libertadores University Foundation, Bogotá, Colombia; 5 Legal Clinic and Conciliation Centre, Los Libertadores University Foundation, Bogotá, Colombia; 6 Department of Public Health, University of Tolima, Ibagué, Colombia; 7 Faculty of Nursing, University of Cartagena, Cartagena, Colombia; 8 Los Libertadores University, Bogotá, Colombia; 9 Department of Social and Human Sciences, Simón Bolívar University, Barranquilla, Colombia; 10 University for Seniors Program, American University of Beirut, Beirut, Lebanon

**Keywords:** Age-friendly cities and communities, Ageism, Colombia, Older adults

## Abstract

Age-friendly cities and communities (AFCC), together with initiatives to combat ageism, are recognised as two of the four action areas of the United Nations (UN) Decade of Healthy Ageing, have received global attention. However, there is a lack of understanding regarding the distinct experiences of older adults in their cities and communities, as well as the extent to which these experiences are linked to their perceptions of ageism. Using a sample of 398 older adults from four cities in Colombia, a latent profile analysis was conducted via the manual three-step method, incorporating demographic covariates and distal outcomes, to identify distinct AFCC profiles among older adults. Three AFCC profiles emerged from the analysis: “high AFCC except financial support” (59.5%), “mixed AFCC with low outdoor spaces/buildings and financial support” (31.4%), and “low AFCC except respect/inclusion” (9.1%). Older adults with disabilities had a higher probability of belonging to the “mixed AFCC with low outdoor spaces/buildings and financial support” group, while age, gender, and subjective social status showed no significant association with profile membership. In addition, ageism experiences differed among the three profiles, as older adults with lower AFCC scores reported higher levels of ageism. The results suggested both a potential method effect associated with negative items in the AFCC, as well as differences in the contexts captured by the AFCC and ageism measures, with the former anchored in community environments and the latter spanning broader interpersonal, institutional, and self-directed experiences. In sum, our findings highlight the need to understand the distinct patterns in how older adults perceive their cities and communities, as well as identify those who may be “at risk” in specific domains.

**Supplementary Information:**

The online version contains supplementary material available at https://doi.org/10.1186/s12877-026-07735-w.

Where people live plays a crucial role in healthy ageing by acting as either a barrier or an enabler, influencing people’s access to resources, patterns of behaviours, and overall quality of life in older age. Recognising the significance of supportive environments, the concept of Age-friendly Cities and Communities (AFCC) has emerged as a global initiative aimed at fostering inclusivity and accessibility of environments that enhance opportunities for health, participation, and security, ensuring quality of life and dignity as people age [1]. To provide a structured approach for developing age-friendly environments, the World Health Organization (WHO) introduced the Age-Friendly Cities and Communities framework, which identifies eight key interrelated areas of urban life that affect the well-being and participation of older adults: community support and health services, transportation, housing, social participation, outdoor spaces and buildings, respect and social inclusion, civic participation and employment, and communication and information. Drawing on this framework, the WHO’s Global Network of Age-Friendly Cities and Communities was established to support and expand age-friendly practices worldwide. As of 2024, the network includes 1705 cities and communities across 60 countries, collectively representing a population of over 330 million people (WHO, [2]). AFCC is one of the four action areas of the United Nations Decade of Healthy Ageing (2021–2030) — alongside combating ageism, developing integrated care, and providing long-term care services — for which the WHO Ageing and Health Unit serves as Secretariat [3]. That combating ageism features alongside AFCC as a co-equal action area is not coincidental: entrenched ageist attitudes can obstruct the development of age-friendly environments, while age-unfriendly communities and cities can in turn reinforce and amplify ageist experiences. Meaningful progress on either front therefore requires coordinated action on both.

Ageism – defined as a multi-dimensional concept including stereotypes (cognition), prejudice (affect), and discrimination (behaviours) directed towards people on the basis of their age — can be self-directed (i.e., internalised), interpersonal, or institutional, and may operate consciously or unconsciously [4]. While ageism can affect people of all ages, the majority of research has focused on its impacts on older adults, documenting wide-ranging associations with health and wellbeing outcomes at the individual level, as well as detrimental impacts and substantial economic costs at the societal level [5–7]. Despite growing recognition of ageism’s importance, current research has primarily examined its antecedents and health consequences, neglecting its complex interactions with environmental factors. Gendron and colleagues [8] highlighted this critical gap, arguing that ageism and its interventions should be conceptualised as dynamic interactions within an ecological framework spanning multiple levels — from close personal relationships to institutional and cultural structures — and emphasising the need for coordinated action across these levels to foster a more age-inclusive society.

The relationship between ageism and AFCC is likely bidirectional, operating through both direct and indirect pathways. On the one hand, where policies manifested at the city and community level (e.g., transport) do not meet the needs of older people (e.g., due to a lack of accessibility or affordability) this can itself be considered a form of ageism, i.e., institutional discrimination. For example, van Hoof et al., [9] explored how age-friendly features and potential ageism manifest in two cities in Netherlands employing a qualitative photoproduction approach. They found that age-stereotypes manifested explicitly and implicitly in communication materials (pamphlets placed too high and printed in small fonts), infrastructure (e.g., insufficient crossing times and non-supportive seating), and some public services (e.g., unfamiliar ticket machines and limited payment options). Moreover, some posters and advertisements linked ageing with physical decline, reflecting a form of visual ageism. This suggests that age-unfriendly aspects of the urban environment may represent a form of institutional ageism, which could shape older adults’ experiences of ageism. More recently, Fernández-Ortiz and colleagues [10] examined AFCC perceptions qualitatively across three localities of Bogotá in Colombia with contrasting socioeconomic profiles, finding that ageism emerged as a cross-cutting theme across all AFCC domains — manifesting through inaccessible infrastructure, mistreatment in transport and health services, and exclusion from employment — and was experienced more intensely in localities with greater socioeconomic vulnerability.

Lack of prioritisation of the needs of older people in community life potentially may reflect entrenched, conscious or unconscious ageism (e.g., the belief that older people have little to contribute to society). Rémillard-Boilard et al., [11] examined AFCC experiences in eleven cities across China, Spain, Mexico, Canada, USA, UK, Japan, Belgium, France, Australia, Chile using a multiple case study approach. Across all cases, challenging negative representations of ageing and shifting perceptions of older age emerged as key priorities of the age-friendly movement, suggesting that addressing ageism is closely intertwined with the success of AFCC initiatives. AFCC programmes were also found to increase awareness of older people’s challenges, making their needs more visible and helping to reduce negative perceptions. However, ageism and a broader lack of interest in ageing-related issues remain significant barriers to AFCC development. Overall, tackling ageist attitudes is critical for AFCC success, while expanding AFCCs can, in turn, potentially help reduce ageism.

Some research has also sought to investigate how age-related environmental factors interact and contribute to older adults’ experiences of ageism. For example, Chan et al., [12] examined the relationship between the neighbourhood-built environment and ageism in Hongkong, China, finding that a greater number of parks within a neighbourhood was associated with lower levels of ageism in all areas, while the greater presence of libraries was linked to reduced ageism only in high-income areas. Sixsmith et al., [13] introduced the PlaceAge project, which found that the creation of inclusive and accessible intergenerational spaces and activities in cities and communities can foster social connections and combat ageism.

Despite the recognised policy importance of both AFCC and ageism, as well as growing body of research evidence, the bidirectional relationship between ageism and AFCC has not been clearly established through quantitative empirical evidence. Existing studies of AFCC have primarily taken one of two approaches: developing conceptual frameworks and guiding principles through literature reviews and syntheses (e.g., [14, 15]), or using qualitative methods — including interviews, photo-diaries, and workshops — to explore older adults’ lived experiences in their cities and communities (e.g., Black and Oh, [16], Sixsmith, [13], Woolrych, [17]. While these contributions are valuable, the absence of sufficient quantitative evidence limits efforts to conduct comprehensive assessments across all WHO AFCC domains, quantifying associations between AFCC and ageism, and support data-driven policy decisions and cross-context comparisons.

The recent development of the Age-Friendly Cities and Communities Questionnaire (AFCCQ; [18]) — a validated, cross-culturally adaptable tool designed to assess older adults’ perceptions of their city’s age-friendliness across all domains in the WHO AFCC framework — has opened new possibilities for survey-based quantitative research in this area. Ongoing validation in multiple regions [19–21] lays the groundwork for extending quantitative research on AFCC across diverse national contexts and examining its associations with ageism experiences. Similarly, the WHO Ageism Experiences Scale [22] represents the first measure of ageism designed to comprehensively capture all dimensions of the construct (i.e., stereotypes, prejudice, and discrimination at self-directed, interpersonal, and institutional levels) across diverse country contexts, was recently developed. The availability of both tools for the first time provides a unique opportunity to rigorously and comprehensively examine the relationship between AFCC and ageism experiences, moving beyond the largely qualitative evidence base that has characterised this field to date.

Finally, while AFCC and ageism initiatives are active worldwide, most network member cities are located in high-income countries, such as those in Europe and North America. Researchers have raised concerns about this geographic imbalance and called for greater evidence from underrepresented regions, particularly where limited resources exacerbate vulnerabilities among older adults [15, 17, 23, 24]. Colombia represents a compelling and underrepresented case for this research. It is one of the most unequal countries in Latin America, undergoing profound social changes driven by rapid population ageing and urbanisation. According to the national census Colombia (DANE, [25]), approximately 4 million people (9.1% of the population) are 65 years and older. This demographic transition can create considerable challenges for both older adults and the broader society. Colombia’s older adult population is characterised by high rates of poverty, low educational attainment, and limited access to formal social protection systems [26, 27], all of which may compound vulnerability to both age-unfriendly environments and ageist experiences [10]. At the same time, Colombia presents important cultural and structural features relevant to ageism and AFCC: as a predominantly urban, highly unequal society with a recent history of conflict and displacement, the social and physical environments in which older Colombians age differ markedly from those studied in high-income country contexts (OECD, [28]). Despite the urgency of addressing AFCC created by these factors, AFCC development in Colombia remains at a relatively early stage. Ibagué became Colombia’s first Global Network of Age-friendly Cities and Communities (GNAFCC) member city when it joined the network in 2016, followed subsequently by Bogotá (Chapinero locality) and several other cities including Rionegro, Envigado, and Manizales (WHO GNAFCC, [29]). Among the four cities studied here, Bogotá and Ibagué are GNAFCC members, while Barranquilla and Cartagena have yet to formally adopt the framework. This variation in AFCC engagement across our sample cities, combined with the broader socioeconomic challenges facing older Colombians outlined above, makes the country a particularly informative and timely context in which to examine how older adults experience the age-friendliness of their cities and communities.

The current study therefore aims to profile older peoples’ experiences of AFCC in Colombia using Latent Profile Analysis (LPA), a person-centred statistical technique designed to identify distinct subgroups within a population based on shared patterns of responses [30]. This approach is motivated by qualitative evidence pointing to substantial heterogeneity in how older adults perceive and experience the age-friendliness of their cities and communities [10], while quantitative AFCC research has been limited and has treated older adults as a homogeneous group. LPA addresses this gap by classifying individuals into subgroups that are similar within but meaningfully different across groups, allowing heterogeneity in AFCC experiences to emerge naturally from the data rather than being implicitly assumed absent. Where distinct AFCC profiles are identified, we then examine whether experiences of ageism differ systematically across them. To date, only one study has employed a person-centred approach. Kim et al., [31] utilised AARP Age-friendly Community Survey data from 6670 adults aged 45 and older residing in 14 communities across the USA and identified three types of age-friendly communities: underdeveloped (22.5%), developing (52.8%), and developed (24.7%). Furthermore, some validation studies of AFCCQ have applied hierarchical cluster analysis to detect subpopulations (e.g., Ivan et al., [32], Pavlovski, [21], Wasserman, [33]. Although not identical in approach, these studies support the existence of meaningful AFCC subgroups across context. By addressing both AFCC and ageism — two of the four action areas of the UN Decade of Healthy Ageing (2021–2030) — in an underrepresented country context, our study offers a unique contribution to the evidence base and provides preliminary quantitative evidence on their interaction. In addition, by identifying which profile types are associated with greater ageism exposure, the findings move beyond average associations to provide actionable evidence for targeting age-friendly interventions toward the subgroups and urban contexts where they are most needed — advancing geriatric public health, urban gerontology, and age-friendly policy development in Colombia and comparable middle-income settings.

## Methods

### Measures

#### AFCC indicators

*Age-friendliness of cities and communities* was measured using a Spanish translation and adaptation of the *Age-Friendly Cities and Communities Questionnaire (AFCCQ)* (Dikken, van den Hoven, van Staalduinen, Hulsebosch-Janssen, & Van Hoof, [18]). The original AFCCQ is a 23-item questionnaire developed to measure older adults’ perceptions of the age-friendliness of their city or community across the 8 domains of the AFCC framework and 1 additional financial situation domain. There are 2 items for housing, 4 for social participation, 2 for respect and social inclusion, 2 for civic participation and employment, 2 for communication and information, 5 for community support and health services, 2 for outdoor spaces and buildings, 2 for transportation, and 2 for financial situation. The scale was modified to ensure its relevance for the Colombian context. Specifically, for the 5 items in community support and health services domains, for the original word “care”, we distinguished between health and social care, i.e., for those items, we separated them into two different items: one for health care and another for social care, while keeping the remaining content unchanged. Following expert advice, this was necessary due to the contrasting health/social care systems of the study context. To maintain consistency with the original scale, we calculated the average score of the social care and health care items to represent the original “care” item. It should be noted that this adaptation may affect direct comparability with prior validation studies of AFCCQ. However, the individual items within each domain remain unchanged, and the separation of health and social care services reflects the institutional realities of the Colombian context, consistent with the flexible, context-sensitive approach to AFCC measurement advocated by Dikken et al., [34]. Domain-level internal consistency estimates are reported in Supplementary Table S1A, with all domains exhibited good internal consistency. To verify that this adaptation maintained construct integrity, we examined within-pair correlations between the health care and social care sub-items for each original item, as well as corrected item-total correlations of each sub-item with the overall domain score. These analyses, reported in Supplementary Table S1B, confirmed that all within-pair correlations were strong (*r*s = 0.53–0.84) and all item-total correlations exceeded conventional thresholds (*r*s = 0.50–0.80), supporting the coherence of the adapted domain structure. At the same time, the within-pair correlations also suggested that the health care and social care sub-items, while related, capture meaningfully distinct aspects of community support, which provide further empirical support to the decision to treat them separately in the Colombian context. All item responses are recorded on a 5-point Like-type scale from *totally disagree (-2)* to *totally agree (2)*.

It is worth noting that the phrase “*neither disagree nor agree*” contains a typographical error (i.e., “Ni desacuerdo ni en desacuerdo”). This was identified during data cleaning following completion of data collection and prior to any analysis. Upon identifying this issue, we took several steps to assess the reliability of our results despite this error. First, we contacted all professors and fieldworkers who had reviewed and conducted the survey. To ensure transparency, consistency, and the avoidance of bias, the research team collaboratively developed standardised call scripts, which are available in Supplementary Material. We inquired whether they had noticed the mistranslation of the response option, whether they had taken any corrective actions, and if participants had expressed confusion or hesitation regarding this response. Additionally, we sought to understand whether the response options had been read exactly as listed and whether participants appeared to avoid selecting the mistranslated option. Results showed that professors from three cities (Bogotá, Cartagena, and Barranquilla) didn’t notice the typo, as they and their students naturally read it as it was intended to be presented. The Ibagué professor noticed this issue during the pilot. However, all students from Ibagué reported that they read the option as if it was written correctly and understood its intended meaning as “Neither Disagree nor Agree”. Out of 27 students, 6 noticed the mistake, but all said they understood the intended meaning. Only one student reported older participants asking for clarification and he clarified the correct meaning. All students followed the listed order when reading the response options. Full documentation of the checking process is provided in the Supplementary Materials, and items of the AFCCQ as administered in Colombia, with frequency of ‘Neither Disagree nor Agree’ responses were provided in Supplementary Materials Table S1.

#### Ageism experiences

*Ageism Experiences* was measured using the *WHO Ageism Experiences Scale* (WHO-Ageism-E15, [22]). It is a 15-item scale designed to capture experiences of ageism according to contemporary multi-dimensional definitions of ageism, which includes items on various aspects of ageism: 2 on self-stereotypes, 1 on self-prejudice, 2 on self-discrimination, 2 on interpersonal stereotypes, 2 on interpersonal prejudice, 3 on interpersonal discrimination, and 3 on institutional discrimination. It was developed to meet the need for measures that could be utilized across diverse cultural and country contexts. Some preliminary psychometric evidence has proved its validity and reliability. For example, some recent validation studies indicated that it possesses good psychometric properties [35, 36]. Responses of this scale are recorded on a 5-point Likert-type scale from *strongly agree* to *strongly disagree*, with an option “*don’t know or not applicable”*. Three items were reverse coded, with higher score represented greater ageism. Scale items are available at WHO Ageism Scale Manual (www.aworld4allages.org).

#### Sociodemographic variables

We asked participants to provide their age (in years), gender (male, female, other), education level (Incomplete primary, Primary completed, Incomplete high school, High school completed, Technical, Technology, Bachelor’s or undergraduate degree, Specialisation, Master’s degree or equivalent level, Doctorate or equivalent level), ethnicity (Mestizo, Blanco, Afrocolombiano, Indígena, Raizal, Gitano o Rom, Others/not listed), subjective life stage, household monthly income, disability status (using Washington Group disability 3 as criteria), and subjective social status (MacArthur Scale).

### Procedure

#### Measure translation

The AFCCQ and WHO Ageism Experiences Scale were translated into Spanish following the TRAPD method [37], which is considered the gold standard for translation and adaptation. To ensure contextual appropriateness for Colombia, we implemented an adapted version of this method. The translation process involved two steps: (1) independent translations by a licensed translator and a machine learning-based translation tool and (2) native Spanish-speaking experts with extensive experience in communication with older adults in Colombia evaluated the translations and determined the final version.

#### Recruitment and data collection

Data for this study were collected from older adults aged 60 years and above in four Colombian cities (i.e., Barranquilla, Bogotá, Cartagena, and Ibagué). Participants met the inclusion criteria of being (1) at least 60 years old and (2) able to communicate in Spanish. The four cities were selected to represent diverse geographic regions, population sizes, and levels of AFCC engagement within urban Colombia. Key demographic and contextual characteristics of each city are summarised in Supplementary Table S2. Participants were recruited through community-based organisations, older people’s groups, and HelpAge International’s local partner networks operating in each city. Fieldworkers were students specialising in relevant disciplines who were native Spanish speakers and proficient in communicating with older adults. Prior to data collection, they received training from the University of Edinburgh, covering the project’s background, survey content, ethical considerations, safety protocols, strategies for managing challenging situations, and post-survey procedures. Data collection was conducted through face-to-face structured interviews, with fieldworkers reading each survey item aloud and recording participants’ responses. A pilot study was conducted to ensure the comprehensibility of survey items. Interviewers were supported by local academic staff and representatives from HelpAge International throughout the process. Data were collected using convenience sampling, given the difficulty of establishing a sampling frame for community-dwelling older adults. Item-level missing data rates for the AFCC indicators and ageism scales are reported in Supplementary Table S3.

### Ethics

This study was reviewed and approved by the Psychology, Philosophy and Language Sciences Research Ethics Committee (PPLSREC) at the University of Edinburgh (Reference No. 27-2425/2). All procedures involving human participants were conducted in accordance with the Declaration of Helsinki, relevant national guidelines and regulations, and the British Psychological Society (BPS) Code of Human Research Ethics. Written informed consent to participate was obtained from all participants prior to data collection.

### Statistical procedure

To obtain AFCC profiles, latent profile analyses (LPA) were conducted on nine AFCC domains (i.e., housing, social participation, respect and social inclusion, civic participation and employment, communication and information, community support and health services, outdoor spaces and buildings, transportation, and financial situation). The manual three-step approach in Mplus version 8.7 [38] were used to conduct LPA with covariates and distal outcomes at the same time. This method ensures that the inclusion of these variables does not lead to different latent profile solutions than would be obtained in a model without them [39, 40].

In Step 1, an unconditional LPA was conducted using nine profile indicators (i.e., nine AFCC domains), with covariates (age, gender, subjective social status, functioning status) and distal outcomes (ageism experiences) included as “auxiliary” variables. The probabilities of belonging to each class and the assigned class membership for each participant were saved. In this step, models with one to six classes were tested. Then the best representative model was selected guided by Nylund et al., [41] recommendations, using fit indices and criteria including: 1) lowest values of sample-size adjusted BIC (SABIC; [42], 2) a significant Vuong-Lo-Mendell-Rubin Likelihood Ratio Test (VLMRT; [43] and Bootstrapped Likelihood Ratio Test (BLRT; [44], 3) sample size of the smallest group (above 5%), and 4) interpretability of the classes. Entropy values, though not used to determine the optimal number of profiles, were obtained to evaluate classification accuracy. Values exceeding 0.8 were considered satisfactory. Next, in Step 2, we obtained the logit values for the classification probabilities for the most likely latent class membership of the best representative model we selected in Step 1, then used them to fix the measurement parameters of the latent profiles in Step 3, which additionally incorporated the covariates and distal outcomes. This model in Step 3 allows for the examination of how covariates affect the AFCC profile membership and test whether distal outcomes (i.e., ageism experiences) differ across AFCC profiles. The Wald test of parameter constraints was conducted to examine whether differences in ageism experiences across the established AFCC profiles were statistically significant. In the LPA, the nine AFCC domain indicators and the four distal ageism outcomes were modelled as continuous variables because each had at least five response options, and prior research has shown that robust continuous estimation performs well with Likert-type scales [45]. Among the demographic covariates, age and subjective social status were treated as continuous, while gender and disability status were treated as binary categorical variables.

Across all analyses, the response option “Neither Disagree nor Agree” was treated as missing due to the typographical error in the survey (see Measures section). Missing data were addressed using Full Information Maximum Likelihood (FIML) in Mplus. To assess robustness, we conducted a series of sensitivity analyses in which the middle response option was retained and recoded as 0, the natural midpoint of the − 2 to + 2 scale. The results of all analyses are reported in the Supplementary Materials (Tables S3, S4, and S5, and Figure S1). Descriptive statistics were computed for both sample characteristics and AFCC domains.

The present sample size falls within the range of *N* ≈ 300–1000 in which fit indices for mixture models have been shown to perform adequately [46]. Additionally, each identified profile was of sufficient size to support meaningful comparison and ensure model stability, with the smallest class comprising 9.1% of the total sample. As in structural equation modelling more broadly, there is no single fixed minimum sample size for LPA; adequacy depends on study-specific conditions including the number of indicators, the degree to which indicators differentiate profiles, profile separation, and relative profile sizes.

## Results

### Descriptive statistics

#### Sociodemographic characteristics of participants

The sample characteristics is presented in Table 1. The sample had a mean age of 72.5 years, ranging from 60 to 95 years. The majority of participants (76%) were female. The sample primarily consisted of three ethnic groups—Mestizo, Blanco, and Afrocolombiano. Data were collected from four cities in Colombia. Most older adults (91%) lacked a bachelor’s degree or equivalent, with 40% not completing primary school. More than half reported a monthly income below COP 500,000. According to the Washington Group Disability3 criteria, 50% of older adults experienced some form of functioning problem.

**Table 1:** Demographic characteristics of participants

Sample characteristics (*N* = 398)	Mean (SD) / *N* (%)
Age	72.50 (7.91)
Gender	
Female	301 (75.63%)
Male	95 (23.87%)
Ethnicity	
Mestizo	165 (41.46%)
Blanco	97 (24.37%)
Ninguno	58 (14.57%)
Afrocolombiano	50 (12.56%)
Indígena	14 (3.52%)
Raizal	1 (0.25%)
Gitano o Rom	1 (0.25%)
Others/not listed	10 (2.51%)
City	
Bogotá	184 (46.23%)
Cartagena	100 (25.13%)
Ibagué	62 (15.58%)
Barranquilla	52 (13.07%)
Education	
Incomplete primary	163 (40.95%)
Primary completed	76 (19.10%)
Incomplete high school	68 (17.09%)
High school completed	27 (6.78%)
Technical	23 (5.78%)
Technology	7 (1.76%)
Bachelor’s or undergraduate degree	9 (2.26%)
Specialisation	4 (1.01%)
Master’s degree or equivalent level	1 (0.25%)
Doctorate or equivalent level	1 (0.25%)
Income	
Less than COP 500,000	232 (58.29%)
COP 500,000 to COP 1,000,000	81 (20.35%)
COP 1,000,001 to COP 2,000,000	58 (14.57%)
COP 2,000,001 to COP 3,000,000	13 (3.27%)
COP 3,000,001 to COP 4,000,000	6 (1.51%)
COP 4,000,001 to COP 5,000,000	2 (0.50%)
COP 5,000,001 to COP 7,500,000	2 (0.50%)
COP 7,500,001 to COP 10,000,000	3 (0.75%)
More than COP 10,000,000	1 (0.25%)
Disability status	
Yes	207 (52.01%)
No	191 (47.99%)
Subjective social status	3.22 (2.09)
Subjective life stage	78.03 (15.59)
Overall ageism experiences	2.48 (0.62)
Self-directed ageism	2.28 (0.73)
Interpersonal ageism	2.35 (0.83)
Institutional ageism	3.10 (0.92)

#### Descriptive statistics of AFCC by cities

The mean and standard deviation (SD) for domain-specific AFCC among older adults in Colombia and across four Colombian cities are shown in Table 2. As shown in the table, older adults tended to rate their cities’ AFCC features in Colombia as neutral. Among specific AFCC domains, civic participation and employment received the highest satisfaction, followed by housing and social participation. Financial support was the most unsatisfactory aspect. These patterns were generally consistent across the cities. Apart from this, Barranquilla demonstrated satisfaction in housing, while Bogotá showed dissatisfaction in community support and health services. Older adults in Cartagena were unsatisfied with outdoor spaces and buildings.

**Table 2:**
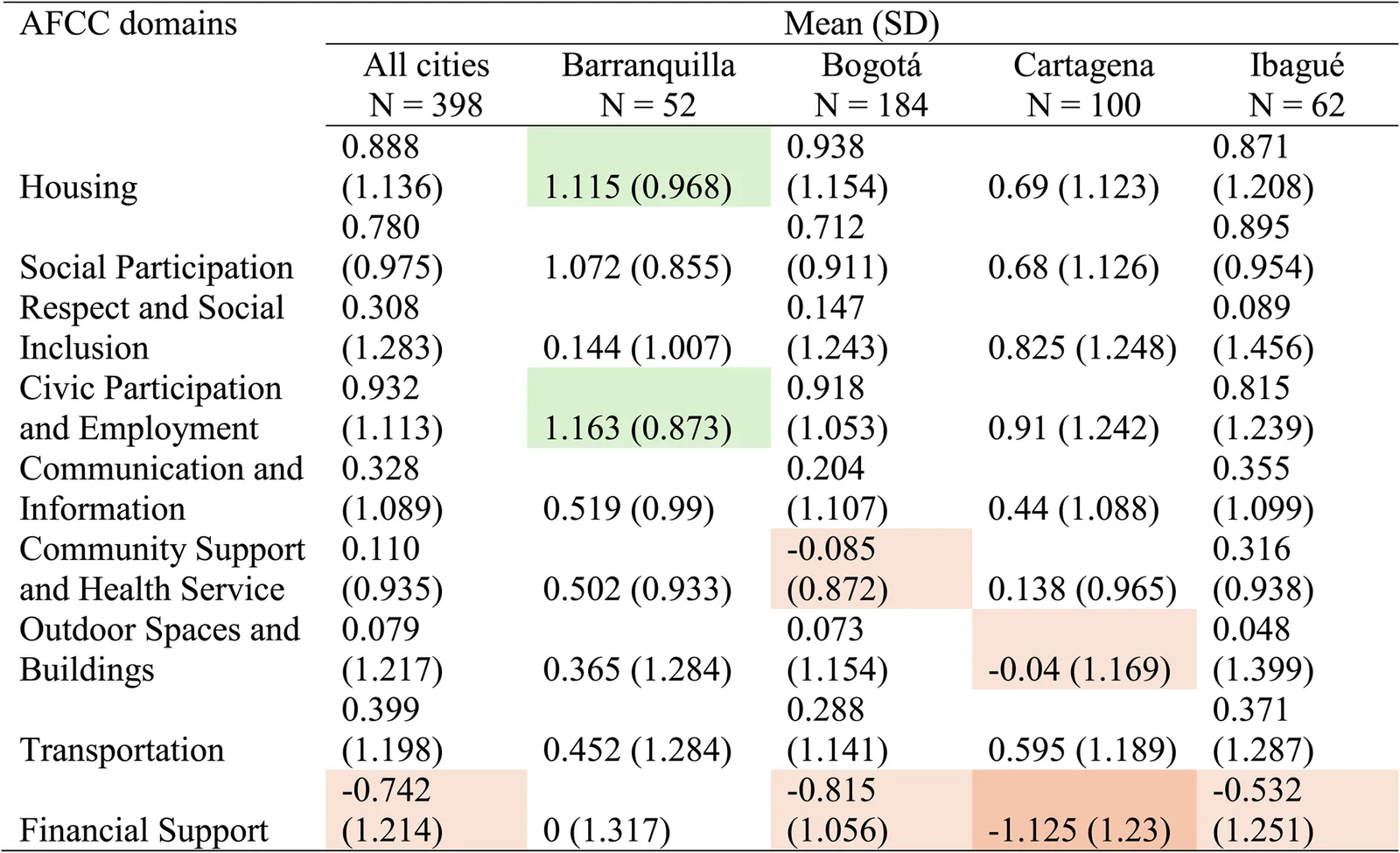
Domain specific AFCC scores by cities in Colombia

The colour zones illustrate older adults' satisfaction levels with the community and city’s age-friendliness, following the colour-coding scheme of the original AFCCQ development paper [34]. Green represents neutral to satisfied perceptions of the AFCC, while orange indicates dissatisfaction. The deeper the colour, the stronger the satisfaction or dissatisfaction level. White cells indicate scores that fall at or close to the neutral midpoint and do not clearly fall within either the satisfied or dissatisfied range. The colour zones reflect the substantive and policy interpretation of scores on the −2 to +2 scale and do not indicate statistical significance

### AFCC best profile identification

Latent profile analysis was performed on the AFCC variables, estimating models ranging from one to six latent profiles; fit indices for all six solutions are presented in Table 3. The BLRT *p* value remained consistently < 0.001 across all solutions, offering limited guidance for determining the optimal number of profiles. The VLMR-LRT displayed a “jump” pattern: the two-profile model was significantly better than the one-profile model, the three-profile model did not reach conventional significance over the two-profile model, and the four-profile model was again significant over the three-profile model. While BIC and SABIC continued to decrease with additional profiles, the magnitude of improvement decreased substantially after three-profile solution. The reductions in BIC and SABIC from one-profile to two-profile, and from two-profile to three-profile, were considerably larger than those observed for any subsequent increment, where improvements became minor. In mixture modelling, selecting the model with the absolute lowest information criteria can lead to overfitting and the extraction of profiles that are statistically separable but substantively trivial [47], and in applied research, parsimony and conceptual interpretability are therefore widely recognised as legitimate criteria alongside statistical fit [48]. After evaluating the deceleration in fit improvement, theoretical interpretability, the size of the smallest subgroup, and model parsimony, the three-profile model was identified as the most suitable choice. Although the VLMR-LRT did not reach conventional significance for the three- over two-profile model (*p* = 0.063), the BLRT was significant (*p* < 0.001), providing statistical support for retaining the third profile. This, combined with an entropy of 0.903 reflecting satisfactory classification accuracy and clear theoretical interpretability of the three-profile solution, supported its selection as the optimal model.

**Table 3:** Fit indices of latent profile model

K	LL	BIC	SABIC	BLRT *p*	VLMR-LRT *p*	Entropy
1	-5578.506	11264.768	11207.653	–	–	–
2	-5275.004	10717.628	10628.783	< 0.001	0.0219	0.947
3	**-5135.484**	**10498.453**	**10377.878**	**< 0.001**	**0.063**	**0.903**
4	-5087.191	10461.731	10309.425	< 0.001	0.0396	0.904
5	-5002.213	10351.64	10167.604	< 0.001	0.6446	0.891
6	-4954.493	10316.065	10100.298	< 0.001	0.0373	0.894

Bold values indicate the preferred latent profile solution retained for subsequent analyses

*K* number of classes, *LL* Model Log Likelihood, *BIC* Baysian Information Criterion, *SABIC* Sample-size Adjusted BIC,* VLMR-LRT p* Vuong-Lo-Mendell-Rubin (VLMR) Likelihood Ratio Test (LRT) p value, *BLRT* Bootstrap Likelihood Ratio Test

Additionally, a sensitivity analysis retaining the middle response option as 0 (the natural midpoint of the − 2 to + 2 scale) confirmed the three-profile solution as optimal (VLMR-LRT *p* = 0.004; entropy = 0.813), with highly consistent profile sizes (Profile 1: 9.2%, Profile 2: 31.5%, Profile 3: 59.3%) and domain patterns (see Supplementary Materials Table S4 and Figure S1).

### Labelling and characteristics of AFCC profiles

**Fig. 1:**
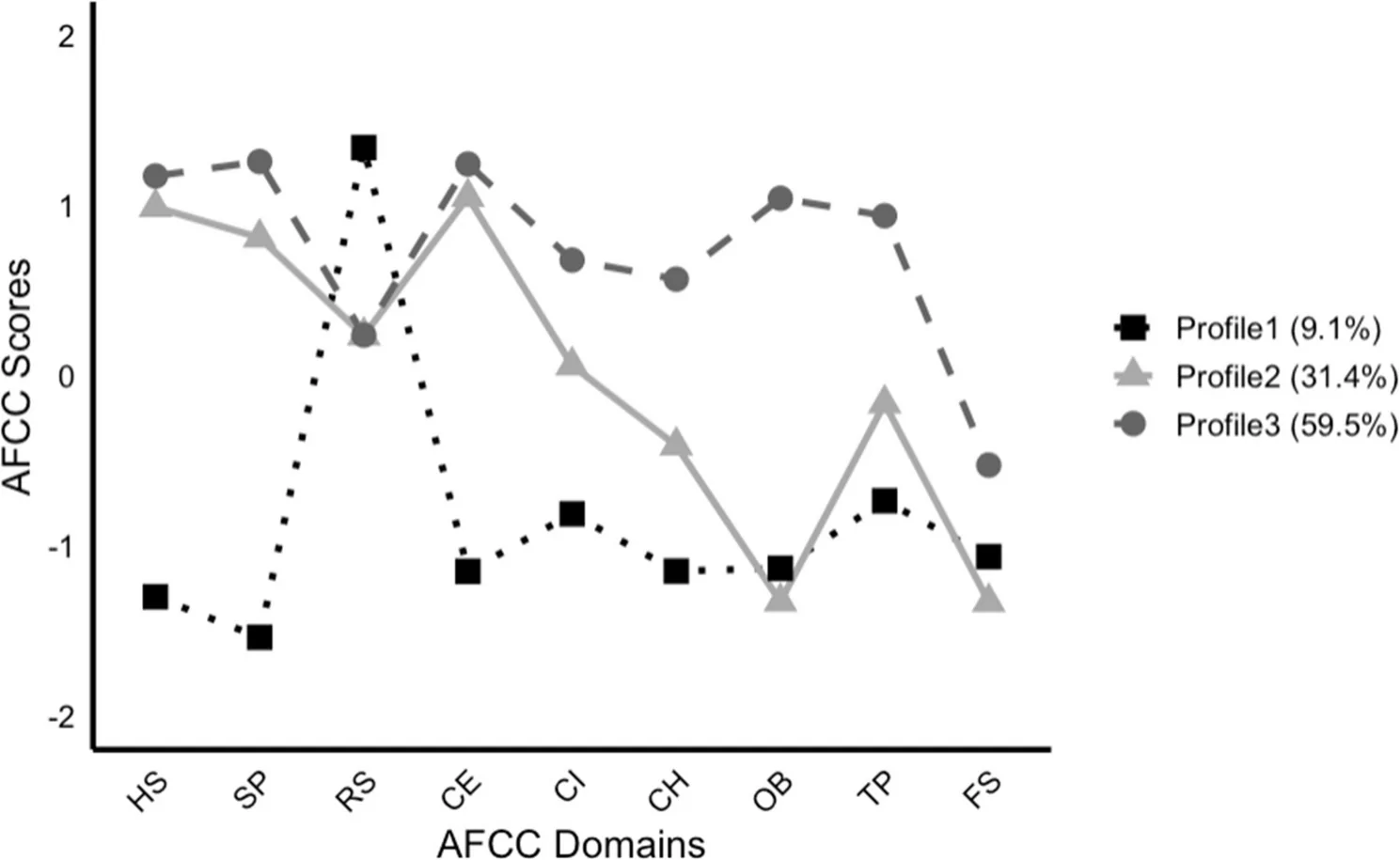
AFCC profiles among Colombian older adults. Note. HS means housing in AFCC, SP means social participation in AFCC, RS means respect and social inclusion in AFCC, CE means civic participation and employment in AFCC, CI means communication and information in AFCC, CH means community support and health service in AFCC, OB means outdoor spaces and buildings in AFCC, TP means transportation in AFCC, FS means financial support in AFCC

As the optimal solution, the three distinct AFCC profiles identified are presented in Fig. 1. The majority of older adults (59.5%) belonged to Profile 3, which exhibited the most positive AFCC experiences across all domains. This profile, labelled as “high AFCC except financial support”, older adults in this profile maintain most positive perception in all domains of their cities and communities, except the respect and social inclusion. 31.4% of older adults belonged to profile 2, which was characterised by a generally neutral perception of their cities and communities. This group also exhibited a distinct decline in the outdoor spaces and buildings domains and reported the lowest perceived financial support among all profiles. Profile 2 then was labelled as “mixed AFCC with low outdoor spaces/buildings and financial support” group. Only 9.1% older adults belonged to profile 1, characterised by generally negative perceptions of their cities and communities across all domains. However, this group reported the highest scores in respect and social inclusion. This profile was labelled as the “low AFCC except respect/inclusion” group.

### Multinomial logistic regression of AFCC profiles on demographic covariates

Demographic covariates and distal ageism experiences variables were simultaneously added to the unconditional model in the subsequent analyses, which refers to the Step 3 described in the statistical analysis plan. First, a multinomial logistic regression was performed as part of Step 3, which examined whether and how covariates (i.e., age, gender, subjective social status, and disability status) were related to AFCC profile membership. Results are shown in Table 4. In sum, age, subjective social status, gender and disability status were not significant associated with the class membership of AFCC, only being disabled was significantly related to a higher probability of belonging to profile 2 (“mixed AFCC with low outdoor spaces/buildings and financial support group”) compared to profile 3 (i.e., “high AFCC except financial support” group), with odds ratio [OR] = 0.489, 95% confidence interval is [CI: 0.288, 0.828].

**Table 4:** Effects of demographic covariates on AFCC profile membership

	Estimates (SE)			
	Age	Subjective social status	Gender^a^	Disability Status^b^
P2 vs. P1^ref^	0.017 (0.024)	-0.176 (0.110)	0.199 (0.530)	0.317 (0.428)
P3 vs. P1^ref^	0.009 (0.022)	-0.062 (0.091)	0.521 (0.498)	-0.399 (0.391)
P3 vs. P2^ref^	-0.009 (0.016)	0.114 (0.074)	0.322 (0.298)	-0.716 (0.269)**

P1 = low AFCC except respect/inclusion; P2 = mixed AFCC with low outdoor spaces/buildings and financial support group; P3 = high AFCC except financial support

*p < 0.05; **p < 0.01; ***p < 0.001

^a^ Reference category is female; ^b^ reference category is “no”

The sensitivity analysis largely replicated the covariate results, with disability status again emerging as a significant predictor of profile membership between profile 2 and profile 3. However, some differences were observed: subjective social status showed significant associations with profile membership in the sensitivity analysis, with higher subjective social status being associated with a higher probability of belonging to both “low AFCC except respect/inclusion” profile and “high AFCC except financial support” profile relative to the “mixed AFCC with low outdoor spaces/buildings and financial support” profile. Additionally, male older adults showed a significantly higher probability of belonging to the “high AFCC except financial support” profile compared to the “mixed AFCC with low outdoor spaces/buildings and financial support” profile. Full results are presented in Supplementary Table S5.

### Differences in ageism experiences across AFCC profiles

Estimates of ageism scores across the three AFCC profiles, and the significance of group differences assessed using Wald’s test, are presented in Table 5. Older adults in profile 1 (i.e., “low AFCC except respect/inclusion” group) experienced the highest ageism experiences at global, self-directed and institutional directed levels, while older adults in profile 2 (i.e., “mixed AFCC with low outdoor spaces/buildings and financial support” group) experienced the highest interpersonal ageism. Older adults in profile 3 (i.e., “high AFCC except financial support” group) experienced the lowest ageism. The Wald test suggested that all levels of ageism experiences scores in profile 3 were significantly lower than those in profile 2, with *p*s < 0.005. Overall and self-directed ageism experiences scores in profile 3 were significantly lower than those in profile 1, with *p*s < 0.005, with no significant differences were found for interpersonal and institutional ageism. The ageism experiences scores between profile 1 and profile 2 were not statistically significant.

**Table 5:** Estimates of ageism scores and pairwise wald tests across three AFCC profiles

	Estimates (SE)			Wald statistics (*p* value)		
	P1	P2	P3	P2 vs. P1	P3 vs. P1	P3 vs. P2
Overall ageism score	2.709 (0.134)	2.627 (0.059)	2.363 (0.040)	0.308 (0.579)	**6.205 (0.013)**	**12.442 (< 0.001)**
Self-directed ageism	2.627 (0.163)	2.373 (0.073)	2.180 (0.046)	1.949 (0.163)	**6.986 (0.008)**	**4.560 (0.033)**
Interpersonal ageism	2.534 (0.183)	2.553 (0.082)	2.225 (0.054)	0.008 (0.929)	2.648 (0.104)	**10.062 (0.002)**
Institutional ageism	3.265 (0.135)	3.257 (0.101)	2.997 (0.059)	0.002 (0.963)	3.292 (0.070)	**4.422 (0.036)**

Bold values indicate statistically significant Wald statistics

P1 = low AFCC except respect/inclusion; P2 = mixed AFCC with low outdoor spaces/buildings and financial support group; P3 = high AFCC except financial support

The sensitivity analysis replicated these patterns. Profile 3 continued to show significantly lower ageism scores than Profile 2 across all four dimensions (overall, self-directed, interpersonal, and institutional ageism). Compared to Profile 1, Profile 3 showed significantly lower overall and self-directed ageism, consistent with the main analysis; notably, interpersonal ageism also reached significance in the sensitivity analysis. No significant differences were found between Profile 1 and Profile 2 on any ageism dimension. Full results are presented in Supplementary Table S6.

## Discussion

The current study aimed to identify distinct latent profiles of AFCC experiences among older adults in Colombia and to examine whether ageism experiences differed across these profiles. The findings provided evidence for three distinct profiles of AFCC domains. The majority − 59.5% – of older adults belonged to the “high AFCC except financial support” profile. Those in this profile hold a mostly positive perception in all domains of their cities and communities, except the respect and social inclusion. Next, 31.4% of older adults belonged to “mixed AFCC with low outdoor spaces/buildings and financial support” group, in which older adults hold relatively neutral perception of their cities and communities, except for the outdoor spaces and buildings domains and financial support domain, which was lowest for this profile group. Only 9.1% older adults belonged to the “low AFCC except respect/inclusion” group. Older adults in this group generally held negative views of their cities and communities across all domains, however, reported the highest levels of respect and social inclusion. The stability of these profiles was further supported by a sensitivity analysis treating the mistranslated middle response option as a valid neutral score (0) rather than missing, which replicated the three-profile solution and yielded consistent findings with respect to both demographic predictors and ageism outcomes across profiles. Overall, this profiling result is consistent with qualitative evidence from Bogotá, where Fernández-Ortiz et al., [10] found that transport, health services, and outdoor spaces were the most problematic AFCC domains— mirroring the low scores in those domains observed in our profiles.

The analysis identified only one significant association between demographic covariates and class membership, showing that individuals with disabilities had a significantly higher probability of being classified in the “mixed AFCC with low outdoor spaces/buildings and financial support” group rather than the “high AFCC except financial support” group. Specifically, the older adults in “mixed AFCC with low outdoor spaces/buildings and financial support” group exhibited relatively low scores in communication and information, community support and health service. They also reported the lowest outdoor spaces and buildings, and financial support among all the profiles. This result highlighted the significant challenges that older people with disabilities faced on those domains, which was further supported by our sensitivity analysis.

Regarding the levels of ageism experience across three AFCC profiles, it was perhaps counterintuitive that the “low AFCC except respect/inclusion” group exhibited the highest levels of ageism experiences, despite having the highest score in AFCC “respect and social inclusion” domain, suggesting a more positive perception of respect and inclusion in their cities and communities. This finding is particularly interesting given that the two items measuring respect and social inclusion in AFCC (i.e., “I sometimes receive annoying or negative remarks because of my age” and “I sometimes face discrimination because of my age”) actually assess self-directed ageism experiences.

To gain further insight into these seemingly contradictory findings, a deeper examination of the AFCC items revealed that the two items measuring the “respect and social inclusion” domain were the only negative items, which may clarify why this discrepancy occurred. In fact, the method effects associated with the negatively worded/negative items are well-recognised and have frequently been found to influence responses in self-reported scales and surveys [49–52]. The general rationale is that concerns about response biases (such as acquiescence, affirmation, or agreement bias) have led researchers to include both positively worded/positive and negatively worded/negative items in scales. However, this approach may instead introduce additional method effects and affect the factorial validity, overestimate or underestimate the relationships between variables, and making the research conclusions inaccurate and less reliable. In our sample, 9.1% of older adults appeared to exhibit this method effect, reporting higher respect and social inclusion in AFCC while simultaneously experiencing the highest levels of ageism. Although these two items are not strictly “negatively worded” but rather “negative” items—measuring a negative aspect rather than using negative phrasing—the method effect may still be present. To assess the reliability of the established profiles, particularly in other domains, a sensitivity analysis was conducted using the same LPA process but excluding the “respect and social inclusion” domain. The results are presented in Supplementary Material Figure S2. The analysis confirmed that the three-class solution was still optimal, with membership classification and domain patterns remaining consistent. As a result, the findings were considered reliable.

A complementary and not mutually exclusive possibility is that the two scales tap meaningfully different social contexts, which may independently contribute to the observed pattern in Profile 1 (“low AFCC except respect/inclusion”). Although the AFCC Respect and Social Inclusion items are not explicitly restricted to the public environment in their wording, they are embedded within a questionnaire framed around older adults’ experiences of their cities and communities. Participants may therefore interpret these items as referring to age-related treatment within the urban or community environment — including public spaces, services, and facilities — rather than in their personal relationships or inner self-perceptions. This questionnaire-level contextual framing effect is consistent with evidence from survey methodology that response context shapes the interpretation of ambiguous items [53]. The WHO Ageism Experiences Scale which was used to measure ageism, by contrast, is framed as a general measure capturing self-directed, interpersonal, and institutional ageism across all life contexts, dimensions that extend well beyond what the AFCCQ was designed to assess. Consistent with Gendron and colleagues’ [8] ecological framework, which conceptualises ageism as operating across multiple interacting yet conceptually distinct levels of the social environment — the AFCCQ Respect and Social Inclusion domain and the WHO Ageism Experiences Scale may be capturing ageism at different ecological levels. The AFCCQ domain maps onto the microsystem, reflecting older adults’ direct perceptions of age-based treatment within their immediate community environment, while the WHO scale spans the microsystem, mesosystem, and exosystem simultaneously. Although these levels interact, Gendron and colleagues’ [8] emphasise that they are also conceptually distinct and may require different forms of intervention. This suggests that the two instruments are not simply measuring the same construct in different instruments, but rather tapping into qualitatively different manifestations of ageism that can coexist and diverge. This may explain why older adults in Profile 1 (“low AFCC except respect/inclusion”) report relatively favourable perceptions of respect in their community context while simultaneously experiencing higher ageism across broader ecological levels. This interpretation finds qualitative support in Fernández-Ortiz and colleagues [10], who found that respect and social inclusion in Bogotá were experienced ambivalently, with neighbourhood ties and community activities providing moments of recognition, while institutional invisibility and everyday ageism persisted, suggesting that positive community-level perceptions and broader experiences of ageism can coexist. Future research employing context-specific measures of ageism would be needed to test this interpretation directly.

Consistent with our findings in a broader sense, previous studies have also reported distinct AFCC profiles (e.g., [21, 32, 33]). In these studies, the authors used the agglomerative (bottom up) hierarchical cluster analysis (HCA) and identified different clusters of AFCC experiences, with five or six clusters identified. Both K-means clustering and LPA are widely used for identifying subgroups within data. However, unlike distance-based clustering technique, the LPA account for measurement error, and provides probabilities of belonging to each latent class, allowing for uncertainty in classification. Additionally, LPA allows for the inclusion of covariates and distal outcomes, enabling researchers to examine antecedents and consequences of class membership. As the AFCCQ is self-reported by older adults, we consider LPA more suitable for profile identification. This study could also serve as a reference for researchers interested in understanding subgroups with different AFCC experiences.

### Strengths and limitations

A particular methodological strength of this study lies in its combined use of two validated, internationally applied instruments — the AFCC Questionnaire and the WHO Ageism Experiences Scale — alongside latent profile analysis, a person-centred approach that moves beyond variable-centred methods to identify distinct subgroups of older adults with meaningfully different patterns of perceived age-friendliness. This methodological framework offers a replicable template for future research seeking to examine how urban environments shape ageism experiences across diverse national and cultural contexts, including longitudinal designs that could illuminate how AFCC profile membership changes over time and whether improvements in age-friendly conditions translate into reductions in ageism at the population level. In addition, our study collected data from older adults in four Colombian cities, a relatively underrepresented region experiencing rapid demographic shifts and urbanisation. According to van Hoof et al., [9], many of the existing AFCC research has been conducted by younger populations (i.e., people who are not older adults evaluate the age-related features of cities and communities). By incorporating direct evidence and experiences from older adults, this study enhances understanding of their circumstances.

The findings may carry direct implications for practice. The identification of “at-risk” subgroups underscores the need for tailored interventions and policies to address their specific needs more effectively. Specifically, our results highlight that older adults with disabilities may face compounded challenges in certain AFCC domains, underscoring the necessity of understanding the needs of those experiencing intersectional disadvantages. The association between lower AFCC profiles and higher ageism further underscores the urgency of prioritising age-friendly investment in under-resourced urban areas.

However, this study is not without limitations. First, errors in response option translation may have influenced the results. Although verification strategies (e.g., contacting professors and fieldworkers) and statistical methods (e.g., treating affected responses as missing values and sensitivity analysis) helped reduce potential bias and confirm the robustness of our results, some degree of uncertainty in the findings and conclusions remains. Moreover, due to sample size limitations (a minimum of 300 participants required for LPA), we were unable to examine AFCC profiles for each of the four cities. Given that cities and communities have distinct landscapes and features, city- and community-level analyses could provide deeper insights into older adults’ experiences. A further concern is the adaptation of the AFCCQ used in this study. The separation of the original description “care” into two distinct items, while substantively justified by the Colombian context, means that direct comparisons of our findings with studies using the standard version should be made with caution. Future validation work examining the psychometric properties of this adapted version in Colombian and broader Latin American samples would strengthen confidence in its measurement integrity. Finally, the representativeness of the current sample warrants consideration. Data were collected using convenience sampling across four Colombian cities — Bogotá, Barranquilla, Cartagena, and Ibagué — which together span distinct geographical regions (Andean, Caribbean, and central Colombia) and represent a range of city sizes and socioeconomic profiles, thereby capturing meaningful diversity within the Colombian urban context. However, several limitations of representativeness should be noted. First, the use of convenience sampling means that participants were not randomly selected, and therefore the data cannot be considered nationally representative.

Second, the gender distribution in our sample — 76% female — is markedly higher than the national older adult population, in which women account for approximately 53–59% of those aged 60 and above according to the SABE Colombia survey, the most comprehensive nationally representative study of Colombian older adults to date [26]. This imbalance likely reflects the well-documented challenges of recruiting older men into health and social research, as well as the community-based recruitment settings used in this study, which tend to attract more women. We should note that the overrepresentation of women in the present sample may influence the interpretation of the identified AFCC profiles. Domains including outdoor spaces and buildings, transportation, social participation, and financial support, are likely to be gender-sensitive. Older women are more likely to experience economic disadvantage, rely on public transport, and face greater safety concerns in public environments, which can constrain mobility and participation [54–56]. Although older women may report stronger motivation for social participation, they may be less likely to engage independently due to these constraints [57]. Although gender was not a statistically significant predictor of profile membership, this does not preclude gendered experiences. Future research in AFCC using more gender-balanced samples or stratified analyses would be valuable, alongside greater attention to intersectional inequalities affecting older women.

Future research replicating these profiles can strengthen the validity of our findings and provide further evidence to test the possibility of methodological effects we hypothesised in the two negative AFCCQ items. Building on earlier insights from Gendron et al., [8], it is essential to address ageism and its interventions across the interacting levels of the ecological system. Therefore, future studies evaluating and monitoring the effects of ageism and AFCC interventions and policies are crucial for translating research into practice and fostering an age-inclusive society. It is also worth noting that the older adult population in Colombia, as elsewhere, is not homogeneous with respect to age itself. The distinction between “young-old” adults and “old-old” adults is relevant in this context, as these subgroups tend to differ in functional capacity, health status, social participation, and vulnerability to exclusion. In Colombia, where the proportion of “old-old” adults is projected to grow substantially over coming decades (DANE, [25]), this distinction has important implications for targeted AFCC policy and intervention. Future research should explicitly adopt age-stratified designs, for example, distinguishing between “young-old” and “old-old” adults, to test whether the AFCC profile structures and their associations with ageism experiences identified here remain stable across different stages of late life, or whether distinct profiles emerge for each subgroup. Such age-stratified evidence would have direct relevance for the design of differentiated, age-sensitive urban policies and AFCC interventions in Colombia and comparable low- and middle-income contexts, where the old-old population is projected to grow most rapidly.

## Conclusions

In conclusion, this study identified three distinct AFCC profiles among older adults in Colombia. General positive perception patterns of AFCC were most prevalent among older adults, while older people with disabilities showed higher probabilities of belonging to the profile that with challenges on “outdoor spaces and buildings” and “financial support” domains. Profiles with overall lower AFCC scores tends to experience higher levels of ageism.

## Supplementary Information

Supplementary Material 1

## Data Availability

Data from this study are available from the research team upon reasonable request.

## Abbreviations

AFCC: Age-friendly cities and communities
AFCCQ: Age-Friendly Cities and Communities Questionnaire
LPA: Latent profile analysis
WHO: World Health Organization
