# Supplementary material for: Latent profiles of the perceived age-friendliness of cities and communities in Colombia: relations to ageism experiences in later life
Source: BMC Geriatr. 2026 Jul 29;26:1349. doi: 10.1186/s12877-026-07735-w (PMC13700173; doi:10.1186/s12877-026-07735-w)
Supplement: Supplementary file 1 [file 12877_2026_7735_MOESM1_ESM.docx]

**Supplementary Materials**

**Table S1A**

Items of the Age-Friendly Cities and Communities Questionnaire (AFCCQ) as Administered in Colombia, with Frequency of ‘Neither Disagree nor Agree’ Responses Treated as Missing

| **Item** | **Item content** | **AFCC Domain** | **n** | **%** |
| --- | --- | --- | --- | --- |
| **Housing (Cronbach’s α = .802)** | | | | |
| Q1 | My house is accessible to me. | Housing | 15 | 3.8 |
| Q2 | My house is accessible to the people who come to visit me. | Housing | 11 | 2.8 |
| **Social Participation (Cronbach’s α = .850)** | | | | |
| Q3 | There are enough opportunities to meet people in my neighbourhood. | Social Participation | 24 | 6.0 |
| Q4 | Activities and events are organised in places that are accessible to me. | Social Participation | 31 | 7.8 |
| Q5 | The information about activities and events is enough for me and also suitable for me. | Social Participation | 44 | 11.1 |
| Q6 | I find the range of events and activities sufficiently varied. | Social Participation | 47 | 11.8 |
| **Respect and Social Inclusion (Cronbach’s α = .791)** | | | | |
| Q7^*^ | I sometimes get annoying or negative remarks because of my age. *[reverse-scored]* | Respect & Social Inclusion | 31 | 7.8 |
| Q8^*^ | I sometimes face discrimination because of my age. *[reverse-scored]* | Respect & Social Inclusion | 31 | 7.8 |
| **Civic Participation and Employment( Cronbach’s α = .745)** | | | | |
| Q9 | I have enough opportunities to interact with younger generations. | Civic Participation & Employment | 27 | 6.8 |
| Q10 | I feel like a valued member of society. | Civic Participation & Employment | 15 | 3.8 |
| **Communication and Information (Cronbach’s α= .605)** | | | | |
| Q11 | Printed and digital information from the municipality and other social institutions is easy to read in terms of font and size. | Communication & Information | 45 | 11.3 |
| Q12 | Printed and digital information from the municipality and other social institutions is written in understandable language. | Communication & Information | 66 | 16.6 |
| **Community Support and Health Services (Cronbach’s α = .903)** | | | | |
| *Note: Each of the five original AFCC items (Q13–Q17) was split into two sub-items distinguishing health care (H) from social care (S), following expert advice regarding the contrasting health/social care systems in Colombia. The domain score for each original item was computed as the mean of its two sub-items, consistent with the original scale scoring.* | | | | |
| Q13H^†^ | The supply of health care in my city is enough for me. | Community Support & Health Services | 52 | 13.1 |
| Q13S^†^ | The supply of social care in my city is enough for me. | Community Support & Health Services | 58 | 14.6 |
| *→ Original Q13: "The supply of care and welfare in my city is enough for me."* | | | | |
| Q14H^†^ | When I am ill, I receive the health care I need. | Community Support & Health Services | 55 | 13.8 |
| Q14S^†^ | When I am ill, I receive the social care and help I need. | Community Support & Health Services | 62 | 15.6 |
| *→ Original Q14: "When I am ill, I receive the care and help I need."* | | | | |
| Q15H^†^ | If necessary, I can easily reach health care services by telephone and in person. | Community Support & Health Services | 60 | 15.1 |
| Q15S^†^ | If necessary, I can easily reach social care and welfare services by telephone and in person. | Community Support & Health Services | 58 | 14.6 |
| *→ Original Q15: "If necessary, I can easily reach care and welfare services by telephone and in person."* | | | | |
| Q16H^†^ | I have enough information about health care services in my neighbourhood. | Community Support & Health Services | 42 | 10.6 |
| Q16S^†^ | I have enough information about social care and welfare services in my neighbourhood. | Community Support & Health Services | 51 | 12.8 |
| *→ Original Q16: "I have enough information about care and welfare services in my neighbourhood."* | | | | |
| Q17H^†^ | Health care workers in my neighbourhood are sufficiently respectful. | Community Support & Health Services | 52 | 13.1 |
| Q17S^†^ | Social care and welfare workers in my neighbourhood are sufficiently respectful. | Community Support & Health Services | 66 | 16.6 |
| *→ Original Q17: "Care and welfare workers in my neighbourhood are sufficiently respectful."* | | | | |
| **Outdoor Spaces and Buildings (Cronbach’s α = .798)** | | | | |
| Q18 | My neighbourhood is sufficiently accessible for a wheeled walker or wheelchair. | Outdoor Spaces & Buildings | 45 | 11.3 |
| Q19 | The shops in my neighbourhood are sufficiently accessible with a wheeled walker or wheelchair. | Outdoor Spaces & Buildings | 63 | 15.8 |
| **Transportation (Cronbach’s α = .741)** | | | | |
| Q20 | I can easily get on the bus or tram in my neighbourhood. | Transportation | 39 | 9.8 |
| Q21 | The bus and tram stops in my neighbourhood are easy to reach and use. | Transportation | 40 | 10.1 |
| **Financial Support (Cronbach’s α = .921)** | | | | |
| Q22 | My income is sufficient to cover my basic needs without any problems. | Financial Support | 37 | 9.3 |
| Q23 | I live well on my income. | Financial Support | 63 | 15.8 |

*Note.* N = 398 participants. n = number of responses to each item coded as missing due to the typographical error in the Spanish translation of the middle response option ("Ni desacuerdo ni en desacuerdo"). % = percentage of 398 participants selecting this option for a given item. All items are scored on a 5-point scale from −2 (Totally Disagree) to +2 (Totally Agree). Missing data were handled using Full Information Maximum Likelihood (FIML) in Mplus.

* Items Q7 and Q8 are reverse-scored prior to domain mean computation, such that higher scores reflect greater age-friendliness.

† Items marked with † represent the Colombian adaptation of the original AFCCQ Community Support and Health Services domain. Each of the five original items (Q13–Q17) was expanded into two sub-items — one pertaining to health care and one to social care — following expert advice and in consultation with the AFCCQ developer, to reflect the distinct health/social care systems in Colombia. Domain scores were computed as the mean of the two corresponding sub-items to maintain consistency with the original scale scoring.

**Table S1B**

*Within-Pair Correlations Between Health Care and Social Care Sub-Items and Corrected Item-Total Correlations with the Community Support and Health Services Domain Score*

| **Original item** | **Sub-item type** | **H–S *r*** | **Item-total *r***  **(corrected)** | **Item-total *r***  **(item dropped)** |
| --- | --- | --- | --- | --- |
| Q13: Supply of care | Health care (afccq-13) | .695 | .646 | .600 |
|  | Social care (afccq-14) |  | .765 | .722 |
| Q14: Care when ill | Health care (afccq-15) | .734 | .750 | .714 |
|  | Social care (afccq-16) |  | .783 | .745 |
| Q15: Reach services | Health care (afccq-17) | .840 | .795 | .736 |
|  | Social care (afccq-18) |  | .790 | .735 |
| Q16: Information | Health care (afccq-19) | .533 | .661 | .632 |
|  | Social care (afccq-20) |  | .712 | .682 |
| Q17: Worker respect | Health care (afccq-21) | .699 | .562 | .495 |
|  | Social care (afccq-22) |  | .560 | .496 |

**Note.** *H–S r* = Pearson correlation between the health care and social care sub-items within each original item pair. Item-total *r* (corrected) = corrected item-total correlation of each sub-item with the full 10-item domain score (correlation with the rest of the scale, item included). Item-total *r* (item dropped) = corrected item-total correlation with the item excluded from the domain score. All analyses used pairwise complete observations.

**Table S2**

Demographic and age-friendly characteristics of the four study cities

| **Characteristic** | **Bogota** | **Barranquilla** | **Cartagena** | **Ibague** | **Notes** |
| --- | --- | --- | --- | --- | --- |
| **Geographic region** | Andina | Caribe | Caribe | Andina | -- |
| **Total population, 2020 (departmental)** | 8,380,801 | 2,600,904 (Atlantico dept.) | 2,219,139 (Bolivar dept.) | 1,427,427 (Tolima dept.) | Bogota D.C. is a special capital district; other figures are department-level and include rural areas. |
| **Population aged 65+ (%, 2020, departmental)** | 9.1% | 8.0% (Atlantico) | 7.8% (Bolivar) | 10.3% (Tolima) | Calculated from age-group projections (65-69, 70-74, 75-79, 80+) as % of total departmental population. |
| **GNAFCC membership** | Yes (Chapinero locality) | No | No | Yes (2016) | Ibague was Colombia's first GNAFCC member (300th globally) in 2016. Exact year of Chapinero membership not confirmed in available sources. |

*Note*. GNAFCC = Global Network for Age-friendly Cities and Communities. DANE = Departamento Administrativo Nacional de Estadistica. Department-level population data are used for Barranquilla (Atlantico), Cartagena (Bolivar), and Ibague (Tolima) as city-level disaggregated age data by single year were not available in the DANE projection files (1985-2020). Bogota D.C. figures refer to the special capital district directly.

**Table S3**

Distributions of the responses of AFCCQ and WHO Ageism Experiences Scales

| Item | Strongly disagree (%) | Disagree  (%) | Neither agree nor disagree  (%) | Agree  (%) | Strongly agree  (%) |
| --- | --- | --- | --- | --- | --- |
| afccq_1 | 10.6 | 10.1 | 3.8 | 41.2 | 34.4 |
| afccq_2 | 7.8 | 6.5 | 2.8 | 45 | 37.9 |
| afccq_3 | 8.3 | 4.3 | 6 | 41.2 | 40.2 |
| afccq_4 | 8.8 | 7.8 | 7.8 | 44 | 31.7 |
| afccq_5 | 10.3 | 8.5 | 11.1 | 47.2 | 22.9 |
| afccq_6 | 8.5 | 12.6 | 11.8 | 39.2 | 27.9 |
| afccq_7 | 25.4 | 29.1 | 7.8 | 24.6 | 13.1 |
| afccq_8 | 28.4 | 26.4 | 7.8 | 24.1 | 13.3 |
| afccq_9 | 10.8 | 10.3 | 6.8 | 39.9 | 32.2 |
| afccq_10 | 8.5 | 3.8 | 3.8 | 32.9 | 51 |
| afccq_11 | 12.1 | 19.3 | 11.3 | 38.2 | 19.1 |
| afccq_12 | 13.6 | 13.8 | 16.6 | 38.4 | 17.6 |
| afccq_13 | 18.8 | 22.6 | 13.1 | 33.7 | 11.8 |
| afccq_14 | 20.6 | 20.1 | 14.6 | 33.7 | 11.1 |
| afccq_15 | 14.3 | 13.6 | 13.8 | 39.9 | 18.3 |
| afccq_16 | 19.3 | 15.6 | 15.6 | 37.4 | 12.1 |
| afccq_17 | 23.9 | 18.3 | 15.1 | 33.2 | 9.5 |
| afccq_18 | 21.6 | 22.1 | 14.6 | 33.2 | 8.5 |
| afccq_19 | 21.1 | 17.6 | 10.6 | 36.9 | 13.8 |
| afccq_20 | 23.9 | 18.6 | 12.8 | 32.2 | 12.6 |
| afccq_21 | 12.3 | 8 | 13.1 | 41 | 25.6 |
| afccq_22 | 13.1 | 7.3 | 16.6 | 42 | 21.1 |
| afccq_23 | 20.9 | 15.3 | 11.3 | 37.4 | 15.1 |
| afccq_24 | 17.6 | 18.8 | 15.8 | 36.2 | 11.6 |
| afccq_25 | 15.3 | 12.1 | 9.8 | 42 | 20.9 |
| afccq_26 | 17.1 | 10.6 | 10.1 | 41 | 21.4 |
| afccq_27 | 39.9 | 28.6 | 9.3 | 15.6 | 6.5 |
| afccq_28 | 33.4 | 29.9 | 15.8 | 13.6 | 7.3 |

| Item | Strongly disagree  (%) | Disagree  (%) | Neither agree nor disagree  (%) | Agree  (%) | Strongly agree  (%) | Don't know / Not applicable  (%) |
| --- | --- | --- | --- | --- | --- | --- |
| ageism_1 | 3 | 3 | 2.8 | 23.6 | 66.3 | 1.3 |
| ageism_2 | 39.9 | 31.9 | 5.5 | 10.8 | 8 | 3.8 |
| ageism_3 | 48 | 31.7 | 2.5 | 5.5 | 8.8 | 3.5 |
| ageism_4 | 19.3 | 30.7 | 12.1 | 23.4 | 11.6 | 3 |
| ageism_5 | 16.6 | 21.1 | 10.3 | 38.4 | 12.1 | 1.5 |
| ageism_6 | 25.6 | 27.4 | 5.8 | 23.1 | 10.1 | 8 |
| ageism_7 | 11.6 | 11.6 | 3 | 38.2 | 32.7 | 3 |
| ageism_8 | 33.2 | 28.1 | 7.5 | 17.3 | 7.8 | 6 |
| ageism_9 | 35.4 | 29.6 | 8 | 15.6 | 6.8 | 4.5 |
| ageism_10 | 32.7 | 29.1 | 7 | 19.8 | 9 | 2.3 |
| ageism_11 | 34.4 | 29.9 | 6.3 | 18.8 | 8.3 | 2.3 |
| ageism_12 | 37.4 | 29.6 | 7.3 | 13.8 | 8 | 3.5 |
| ageism_13 | 17.6 | 11.3 | 8.3 | 25.1 | 31.7 | 6 |
| ageism_14 | 14.3 | 9 | 14.8 | 29.1 | 28.6 | 4 |
| ageism_15 | 15.8 | 14.8 | 8.3 | 24.1 | 30.2 | 6.8 |

**Table S4**

Sensitivity Analysis: Fit Indices of Latent Profile Model Treating Middle Option As 0

| K | LL | BIC | SABIC | BLRT *p* | VLMR-LRT *p* | Entropy |
| --- | --- | --- | --- | --- | --- | --- |
| 1 | -5494.84 | 11097.436 | 11040.321 | – | – | – |
| 2 | -5204.21 | 10576.04 | 10487.195 | <.001 | 0.1939 | 0.939 |
| **3** | **-5070.32** | **10368.124** | **10247.549** | **<.001** | **0.0035** | **0.813** |
| 4 | -5018.294 | 10323.939 | 10171.633 | <.001 | 0.2445 | 0.844 |
| 5 | -4992.917 | 10333.048 | 10149.012 | <.001 | 0.2574 | 0.825 |
| 6 | -4966.185 | 10339.449 | 10123.682 | <.001 | 0.5275 | 0.837 |

*Notes*. K = number of classes; LL = Model Log Likelihood; BIC = Bayesian Information Criterion; SABIC = Sample-size Adjusted BIC; VLMR-LRT *p* = Vuong-Lo-Mendell-Rubin (VLMR) Likelihood Ratio Test (LRT) *p* value; BLRT = Bootstrap Likelihood Ratio Test.

**Table S5**

Sensitivity Analysis: Effects of demographic covariates on AFCC profile membership

|  | Estimates (SE) | | | |
| --- | --- | --- | --- | --- |
|  | Age | Subjective social status | Gender^a^ | Disability Status^b^ |
| P2 vs. P1^ref^ | 0.032 ( 0.025) | -0.315 (0.123)* | 0.057 (0.584 ) | 0.558 (0.442 ) |
| P3 vs. P1^ref^ | -0.007 ( 0.024) | -0.011 (0.096) | 0.847 (0.542) | -0.579 (0.407) |
| P3 vs. P2^ref^ | -0.039 (0.020) | 0.304 (0.096)** | 0.790 (0.362)* | -1.138 (0.313)*** |

*Note*. P1 = low AFCC except respect/inclusion; P2 = mixed AFCC with low outdoor spaces/buildings and financial support group; P3 = high AFCC except financial support; **p* < 0.05; ***p* < 0.01; ****p* < 0.001.

^a^ Reference category is female; ^b^ reference category is “no”.

**Table S6**

Sensitivity Analysis: Estimates of Ageism Scores and Pairwise Wald Tests across Three AFCC Profiles

|  | Estimates (SE) | | | Wald statistics (*p* value) | | |
| --- | --- | --- | --- | --- | --- | --- |
|  | P1 | P2 | P3 | P2 vs. P1 | P3 vs. P1 | P3 vs. P2 |
| Overall ageism score | 2.75 (0.140) | 2.692 (0.058) | 2.294 (0.042) | 0.139 (0.709) | **10.010 (0.002)** | **27.108 (<.001)** |
| Self-directed ageism | 2.671 (0.170) | 2.452 (0.076) | 2.111 (0.047) | 1.331 (0.249) | **10.201 (0.001)** | **12.303 ((<.001)** |
| Interpersonal ageism | 2.602 (0.189) | 2.629 (0.082) | 2.137 (0.058) | 0.016 (0.899) | **5.629 (0.018)** | **19.597 (<.001)** |
| Institutional ageism | 3.235 (0.143) | 3.313 (0.103) | 2.944 (0.065) | 0.191 (0.662) | 3.425 (0.064) | **7.338 (0.007)** |

*Note*. Bold values indicate statistically significant Wald statistics.

P1 = low AFCC except respect/inclusion; P2 = mixed AFCC with low outdoor spaces/buildings and financial support group; P3 = high AFCC except financial support.

**Figure S1**

Sensitivity Analysis: AFCC profiles among Colombian older adults Treating Middle Option As 0

**Figure S2**

Sensitivity check of latent profile analysis without “Respect and Social Inclusion” domain

*Note*. HS means housing in AFCC, SP means social participation in AFCC, RS means respect and social inclusion in AFCC, CE means civic participation and employment in AFCC, CI means communication and information in AFCC, CH means community support and health service in AFCC, OB means outdoor spaces and buildings in AFCC, TP means transportation in AFCC, FS means financial support in AFCC.

**Scripts to call enumerators**

We are writing/calling to follow up on the data collection process for the AFCCQ and Ageism questionnaire. During our recent data review, we identified a potential issue with the translation of one of the response options. Specifically, the phrase “neither disagree nor agree” appears to have been translated as “neither disagree nor disagree” (“Ni desacuerdo ni en desacuerdo”) in the section titled “Cuestionario sobre ciudades y comunidades amigables con las personas mayores” where questions covered topics such as the accessibility of housing, neighborhood facilities, stores, transportation, opportunities to meet new people, adequacy of information and events, situations of discrimination based on age, opportunities to interact with other age groups, and the availability of medical services and care.

To ensure the validity of our findings and understand the potential impact of this translation on the data collection process, we would like your insights on the following points:

1. Do you recall noticing the mistranslation of the response option “neither disagree nor agree” as “neither disagree nor disagree” during the fieldwork?

2. If you noticed the mistranslation, did you resolve it in any way while administering the survey? If yes, what explanation did you provide?

3. Did participants ever ask for clarification about this response option or seem confused by it?

4. When reading out the response options, did you read them in the exact order as listed, including this response? If you noticed the error, how did you address it?

5. Do you recall if participants tended to avoid choosing this response option during the survey?

Your feedback will be invaluable in helping us better understand the implications of this issue and ensure the accuracy of our data. Thank you for your cooperation.

**Briefing on the Consultation with Surveyors**

**LAC Ageism Scale and AFCCs**

To address the typo in the "Neither Disagree nor Agree" option in the AFCC Scale of the survey, we consulted four professors, each representing a university that supported the survey's implementation. Additionally, we reached out to 27 of the 28 students who assisted as surveyors across four cities; only one student has yet to respond.

Three professors provided their input through Microsoft Teams meetings, while one responded via email. All students were consulted via phone calls, and all interactions were recorded with their verbal consent.

A detailed report of each consultation, including responses and a link to the recordings, [is available here.](https://helpage-my.sharepoint.com/:x:/g/personal/nelson_tamayo_helpage_org/Ee1s9x2mqZlCrVy3djyu0GMBtx3gV0wR6IX_ydVHDL9yWg?e=BFL3q2)

Key Conclusions from the Consultation

-	Professors from Bogotá, Cartagena, and Barranquilla stated that they had not noticed the typo until it was pointed out during the consultation. They explained that both they and their students are familiar with this type of question and the Likert scale used in the AFCC section. As a result, they naturally read and understood the option “Neither Disagree nor Agree” as if it had been written correctly. They also noted that the mistake is subtle and easy to overlook.

-	The professor from Ibagué stated that she noticed the typo during the pilot phase and reported it, along with other survey errors, to Red Colombiana. However, the professors observed that the issue remained uncorrected when the survey was administered. That said, all students from Ibagué reported that they read the option as if it was written correctly and understood its intended meaning as “Neither Disagree nor Agree.”

-	Out of 27 students, 6 affirmed they noticed the mistake and 21 affirmed they did not notice it.

-	All 27 students stated that they read the option as if it had been written correctly. They also noted that the options on this scale were familiar and straightforward, leaving no room for confusion. They understood this option as a neutral response and explained it that way to older people.

-	26 students mentioned older people didn’t ask for clarifications on this specific option and didn’t seem to be confused by it. Only one student mentioned that some older participants asked about the option, but she clarified its meaning and read it as if it had been written correctly.

-	All 27 students reported they read the questions and response options in the exact order as listed.

-	7 students mentioned participants didn’t avoid choosing this option. 20 students mentioned participants avoided the option but not because of the mistake, only because they preferred other options and didn’t have a neutral opinion in most of questions.

-	Students also stated that this response option was not confusing for older people. They noted that other aspects, such as the subjective life stage question and the significant variation in scale types throughout the survey, were more challenging for participants.
